# Interaction of microcrystalline chitosan with graphene oxide (GO) and magnesium ions in aqueous solution

**DOI:** 10.1186/s13065-019-0574-y

**Published:** 2019-04-19

**Authors:** Marta E. Lichawska, Aleksander Kufelnicki, Magdalena Woźniczka

**Affiliations:** Department of Physical and Biocoordination Chemistry, Faculty of Pharmacy, Medical University of Łódź, 90-151 Łódź, Poland

**Keywords:** Biomaterials, graphene oxide (GO), Microcrystalline chitosan, Metal-polymer complexes, Equilibria in aqueous solution

## Abstract

**Background:**

Thanks to its specific chemical and physical properties, graphene has aroused growing interest in many fields of Science and Technology. The present study focuses on the properties of microcrystalline chitosan (MCCh): a compound known to increase the biocompatibility of various matrices, including those made of graphene layers, enabling the controlled release of molecules of therapeutic compounds. The study exploits the potential of MCCh to complex with metal ions, in this case Mg^2+^, and attempts to describe such interactions when the system is enriched with graphene oxide (GO). These findings would open completely new areas of knowledge about GO as a drug carrier.

**Results:**

Potentiometric analysis found that in the GO–Mg system, complexes of ML’ type were formed, where M = Mg^2+^; L’ = GO (log *β*_11’0_ = 9.5 (3)) and ML’_2_ (log *β*_12’0_ = 13.2 (4)), whereas in the GO–Mg^2+^–MCCh system, a mixed-type complex MLL’ was also formed, in which L = MCCh: this complex demonstrated the overall stability constants log *β*_111’_ = 11.2 (3) for degree of deacetylation DD 74.4% and log *β*_111’_ = 12.4 (4) for DD 97.7%. FT-IR analysis showed interactions in the GO–Mg^2+^–MCCh (DD = 97.7%) system. In addition, the amide II—NH band was displaced from 1623 cm^−1^ to two bands at 1633 cm^−1^ and 1648 cm^−1^, resulting from the interaction of the metal ion, and the absorption band of the corresponding NH in the chitosan acetyl group was shifted from 1304 to 1351 cm^−1^. When chitosan with a deacetylation degree lower than 74.4% was applied, the amide bands I and II differed only in their intensity. A greater impact on absorption was observed for the acetyl NH group of chitosan, for which the corresponding band shifted from 1319 to 1361 cm^−1^.

**Conclusions:**

The results confirm the ability of GO–Mg^2+^–MCCh to create complex arrangements. It can form a basic complex of one metal ion and one ligand molecule (GO) in the case of ML’ (where L’ = GO), or two molecules of GO with a metal ion M (Mg^2+^) in the case of ML’_2_. A mixed complex of MLL’ type is also formed, with two ligands: L = MCCh with deacetylation degrees DD = 74.4% and 97.7% and graphene oxide L’ = GO. In the latter case, FT-IR spectroscopy was used to confirm the mode of interaction. The GO–Mg^2+^–MCCh system may be used as carrier in modern magnesium containing medicines or as auxiliary substances in pharmacy.

## Introduction

Graphene is characterized by specific chemical and physical properties and has therefore been the subject of a great deal of research regarding its potential applications in Science and Technology. The oxidized derivative of graphene is known as graphene oxide (GO), which is obtained from graphite oxide, also originally referred as GO [1]. GO is used as a substrate in nano-electronics, composite materials, energy technology, sensors and catalysis [2], and is under evaluation for use as a nano-carrier in medications [3].

The presence of certain functional groups makes GO biocompatible, stable in aqueous solution and compatible with polymers such as chitosan. Functional oxygen groups such as hydroxyl (OH), carbonyl (C=O) and carboxyl (COOH) [4] can link to the GO molecule, allowing it to interact with various systems. Graphene oxide itself can be obtained by the oxidation of graphene using a wide variety of compounds, including nitric acid (V), sulfuric acid (VI) and potassium permanganate [5].

**Fig. 1:**
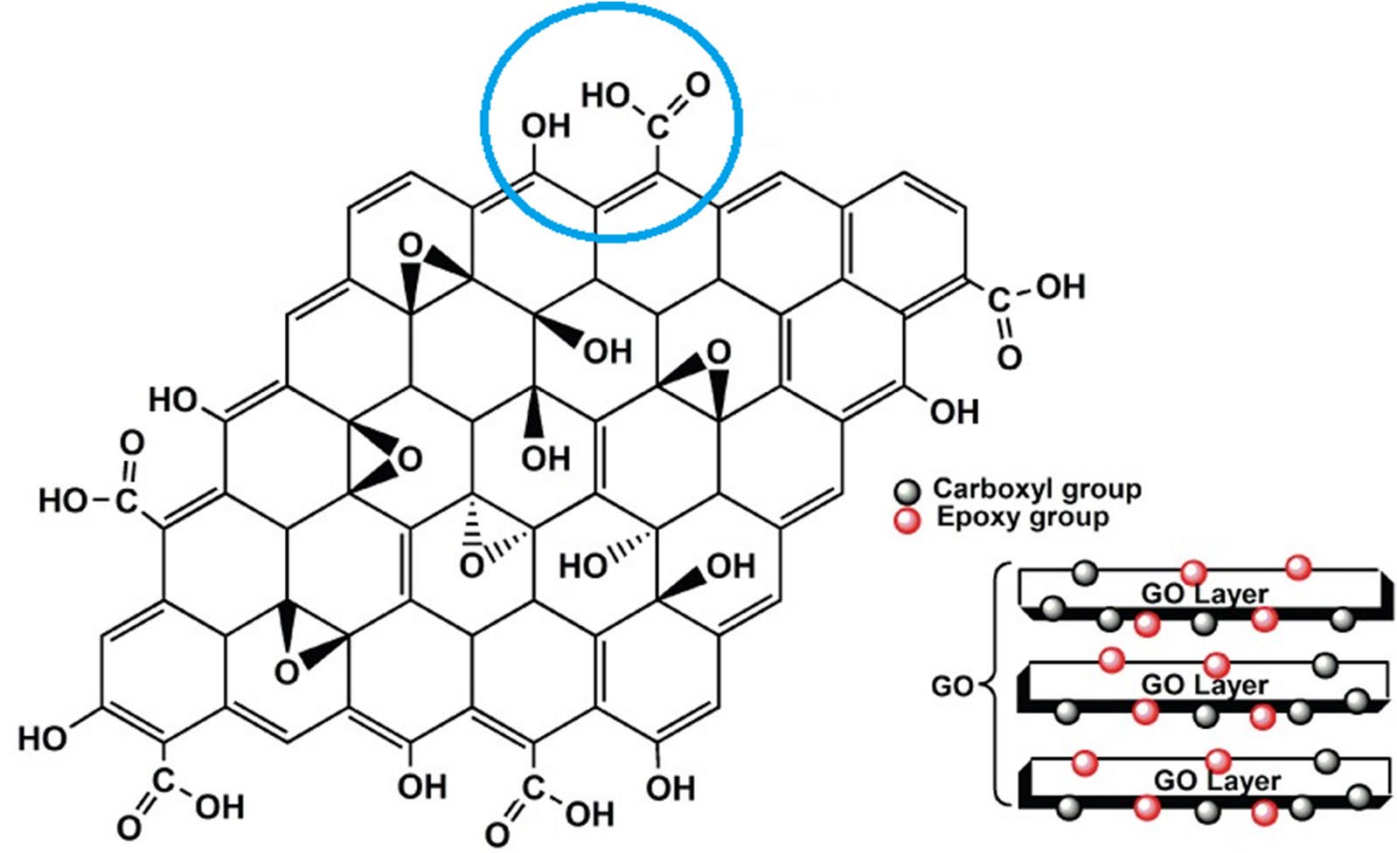
The arrangement rings and functional groups in a single layer of graphene [8]

Although a number of studies have examined the synthesis of graphene and its oxide, relatively little is known of their chemical structures. Such analysis is complicated by the fact that they are non-stoichiometric compounds, and their structure is strictly dependent upon the conditions used during synthesis. Even so, a precise knowledge of their structure is nevertheless essential if these substances are to be used correctly in everyday applications [6]. Graphene is, however, known to be a variant of allotropic carbon, with a two-dimensional structure of monatomic thickness. The carbon atoms form a six-membered hybridized sp^2^ structure resembling a honeycomb [7]. The structure of this material has a significant impact on its properties, which are also influenced by the type and location of functional groups, as shown in Fig. 1 [8].

The latest model of GO has been derived from nuclear magnetic resonance spectroscopy (NMR) [6]. The compound appears to consist of two regions: an aromatic region with non-oxidized benzene rings and an aliphatic region consisting of six-membered rings. Oxygens containing functional groups, such as epoxy and hydroxyl, are located in the basal plane, with carboxyl groups and hydroxyl groups located at the edges. This structure has been confirmed by high-resolution transmission electron microscopy (TEM) [9]. Any further analysis of the interactions of GO presented in this work are based precisely on the structure proposed by Lerf and Klinowski [6].

GO shows a very high ability to accumulate in the lungs, spleen, liver and kidneys, and the lack of biodegradation and the potential for long-term toxicity associated with the presence of oxygen functional groups are major causes for concern when using GO in biomedical applications. Despite this, extensive research has identified ways to reduce toxicity. Some studies indicate the presence of a relationship between GO particle size and hemolytic activity, with smaller particle sizes being associated with greater hemolytic activity and vice versa. Therefore, layering of the analyzed compound by chitosan, a polysaccharide biopolymer composed of d-glucosamine (2-amino-2-deoxy-d-glucose) and *N*-acetyl-d-glucosamine (2-acetamido-2-deoxy-d-glucose), should result in complete elimination of this activity. Such investigations are in progress, although their results are not yet fully clear.

Microcrystalline chitosan (MCCh) is one of the most common modifications of basic chitosan. The polymer exhibits a number of beneficial properties, such as biocompatibility, decomposition into non-toxic products, and broad antiviral, antifungal and antibacterial activity. This aim of the present study is to determine the biocompatibility of MCCh formed from various substrates, including graphene layers, which allow for controlled release of molecules for therapeutic compounds [10]. Due to their great importance to the human body, Mg^2+^ ions were chosen to investigate the potential of the GO + MCCh combination to complex metal ions. Such studies could provide new knowledge of GO as a drug carrier or excipient [11, 12]. Chitosan films treated with GO have already been investigated by Ping Zuo et al. [3] using a range of analytical techniques including X-ray analysis, electron spectroscopy, differential scanning calorimetry and gravimetric analysis; the findings indicate that these films are subject to tensile forces which increase significantly in the presence of GO. In addition, such a system shows greater thermal stability than chitosan. Moreover it remains biocompatible and biodegradable, and possesses good solubility in aqueous systems, similar to chitosan alone.

## Results and discussion

### Complex formation GO with Mg^2+^and GO–Mg–MCCh

**Fig. 2:**
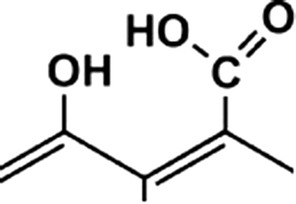
The basic structure of a graphene oxide fragment responsible for coordination of the metal ion

To determine the properties of graphene oxide (GO) with Mg^2+^ ions, it was first necessary to identify the protonation constants of the ligand. The ligand GO was conventionally called a *molecule*, because GO did not display any separate, repeatable mers, unlike the case of typical polymers, which possess an indefinite molecular formula. A molecular unit with an approximate molecular weight of 98.04 was identified according to the Lerf–Klinowski model (Fig. 2), taking into account the nearest vicinity of the functional groups involved in coordination of Mg^2+^. Based on the determined density of the aqueous GO suspension at a given temperature, it was then possible to calculate the mass of GO in a 4 ml volume of the sample, and thus the number of mmol present.

**Fig. 3:**
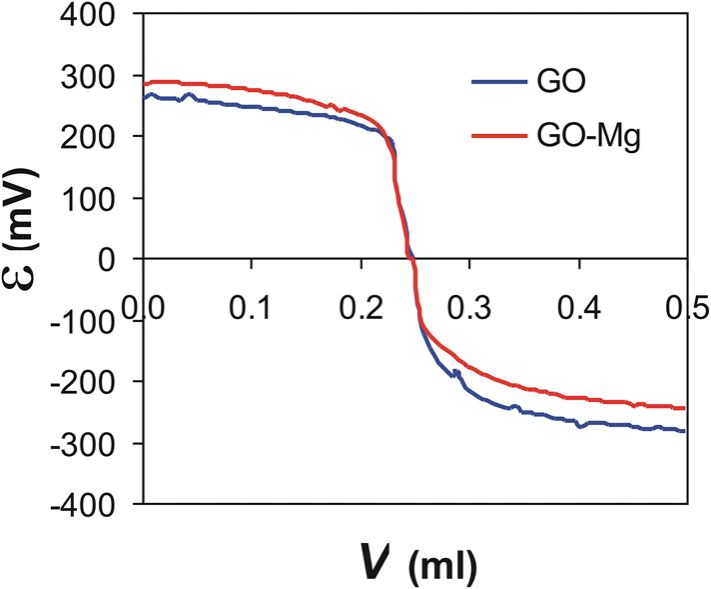
Titration 4 ml samples in an oxide graphene system (GO)–Mg^2+^ in absence of the metal ion (curve 1) and in the presence of Mg^2+^ (curve 2). Total concentration: C_GO_ = 7.75·10^−3^ M; C_Mg_ = 8.75·10^−4^ M

The graph in Fig. 3 showing the relationship between the electromotive force of the cell and the volume of the added base solution indicates an increase of the SEM value for the curve of Mg^2+^ (curve 2); this increase corresponds to reduction in pH due to the complexing deprotonation of functional groups. The calculations allowed two protonation constants for GO to be determined: log *β*_01’1_ = 9.96 (5) for the carboxyl group and log *β*_01’2_ = 12.60 (5) for the hydroxyl group.

**Fig. 4:**
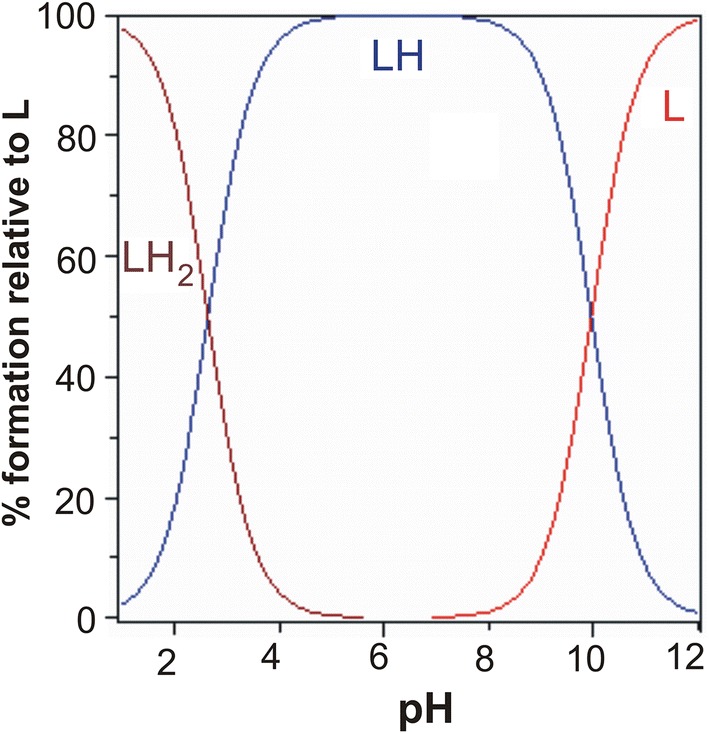
Percentage of protonated GO species as a function of pH. Sample of volume 4 ml contained 0.031 mmol GO and 0.030 mmol of HNO_3_

A graph indicating the percentage of the protonated form (Fig. 4) against pH shows that in an acidic medium, the first proton is dissociated from the carboxyl group (COOH), while a proton from the –OH group is removed together with further alkalization of the solution.

The next stage of the experiment was to determine the stability constants for the system GO–Mg^2+^. The equilibrium model assumes that different possibilities exist for the formation of simple ML complexes, e.g. ML’, ML’_2,_ ML’_3_: the central atom is a metal cation M-Mg^2+^ surrounded by L’-GO ligands. The possible protonated forms ML’H and ML’_2_H are explained by the presence of a central atom with one or two ligands and the simultaneous protonation of the OH-GO group. By matching the proposed theoretical model to the experimental curve, two simple complex types were identified, ML’ and ML’_2_, with the respective formation constants log *β*_11’0_ = 9.5 (3) and log *β*_12’0_ = 13.2 (4), where ML’ indicates a complex involving one metal ion and one GO ligand molecule, and ML’_2_ indicates two GO particles in combination with a metal ion.

**Fig. 5:**
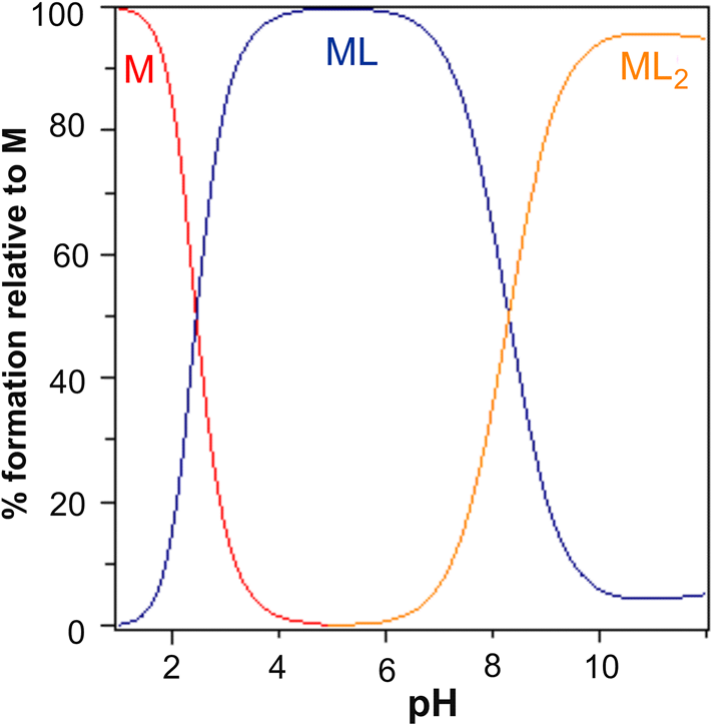
The percentage of the complexes formed in the GO–Mg system

The simple complex type (ML) reaches a maximum at around pH 5 (Fig. 5). The share of the ML’_2_ complex increases as alkalization continues, with a maximum level reached around pH 11.

The determined stability constants for the GO–Mg^2+^ system were used for the equilibrium model of the heteroligand system MCCh–Mg^2+^–GO. At this stage, MCCh with two degrees of deacetylation (DD = 74.4% and 97.7%) was applied. An initial log *β*_111_’ value of 14.0 (MLL’) for the mixed complex was established (Table 1).

**Table 1:** The constants used in the calculations for heteroligand complexes

log *β*_011_ = 6.50 (LH)	Protonation constant of MCCh [13, 14]
log *β*_01_’_1_ = 9.96 (L′H)	The first protonation constant of GO for the OH^−^ group, determined under the conditions of the current experiment
log *β*_01_’_2_ = 12.60 (L′H_2_)	The second protonation constant of GO for the COO^−^ group, determined under the conditions of the current experiment
log *β*_00-1_ = − 13.77 (H^−1^)	pK_w_
log *β*_10-1_ = − 11.70 (MH^−1^)	Hydrolysis constant of Mg^2+^ [15]
log *β*_110_ = 3.29 (6) (ML)	The value obtained from previous tests for the system MCCh–Mg^2+^ [16]
log *β*_111_’ = 14.0 (MLL’)	In this case, it was assumed that the central atom Mg^2+^ is surrounded by the ligand molecule L-MCCh and ligand L′-GO

In the case of the mixed complexes comprising two ligands, i.e. GO and MCCh of DD 74.4% or 97.7% and a metal ion, single titration curves were characterized by a local discontinuity, leading to a greater standard deviation for each formation constant (Table 2).

**Table 2:** Summary of the formation constants of simple and mixed complexes

	GO–Mg^2+^	GO–Mg^2+^–MCCh SD 74.4%	GO–Mg^2+^–MCCh SD 97.7%
log *β*_11_’_0_	9.5 (3)		
log *β*_12_’_0_	13.2 (4)		
log *β*_111_’		11.2 (3)	*β*_111_’ = 12.4 (4)

**Fig. 6:**
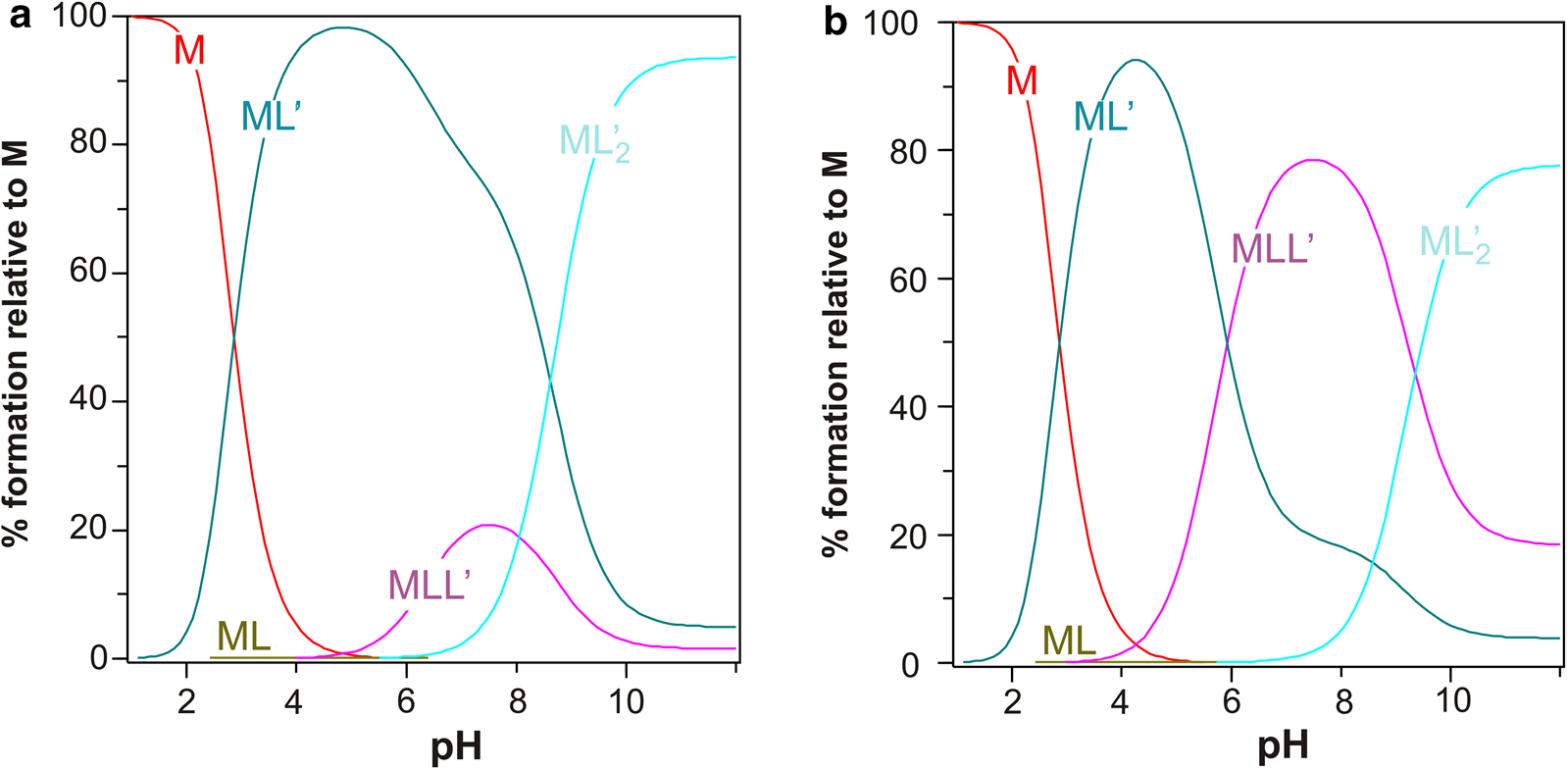
Simulated percentage of the formed complexes as a function of pH for the GO–Mg^2+^–MCCh system. The MCCh gel of deacetylation degree DD = 74.4% (**a**) and 97.7% (**b**), respectively. Program HySS

The simulated species distribution diagram of the formed complexes in the GO–Mg^2+^–MCCh system (Fig. 6) showed the presence of the following complexes : ML, ML’, ML’_2_, MLL’ (with a maximum level at about pH 8.5), ML’_2_ (l-chitosan, L’-GO, M-Mg^2+^). MCCh only constituted a very small share of the ML complex, contrary to the ML’ GO complex.

### Infrared spectra

**Fig. 7:**
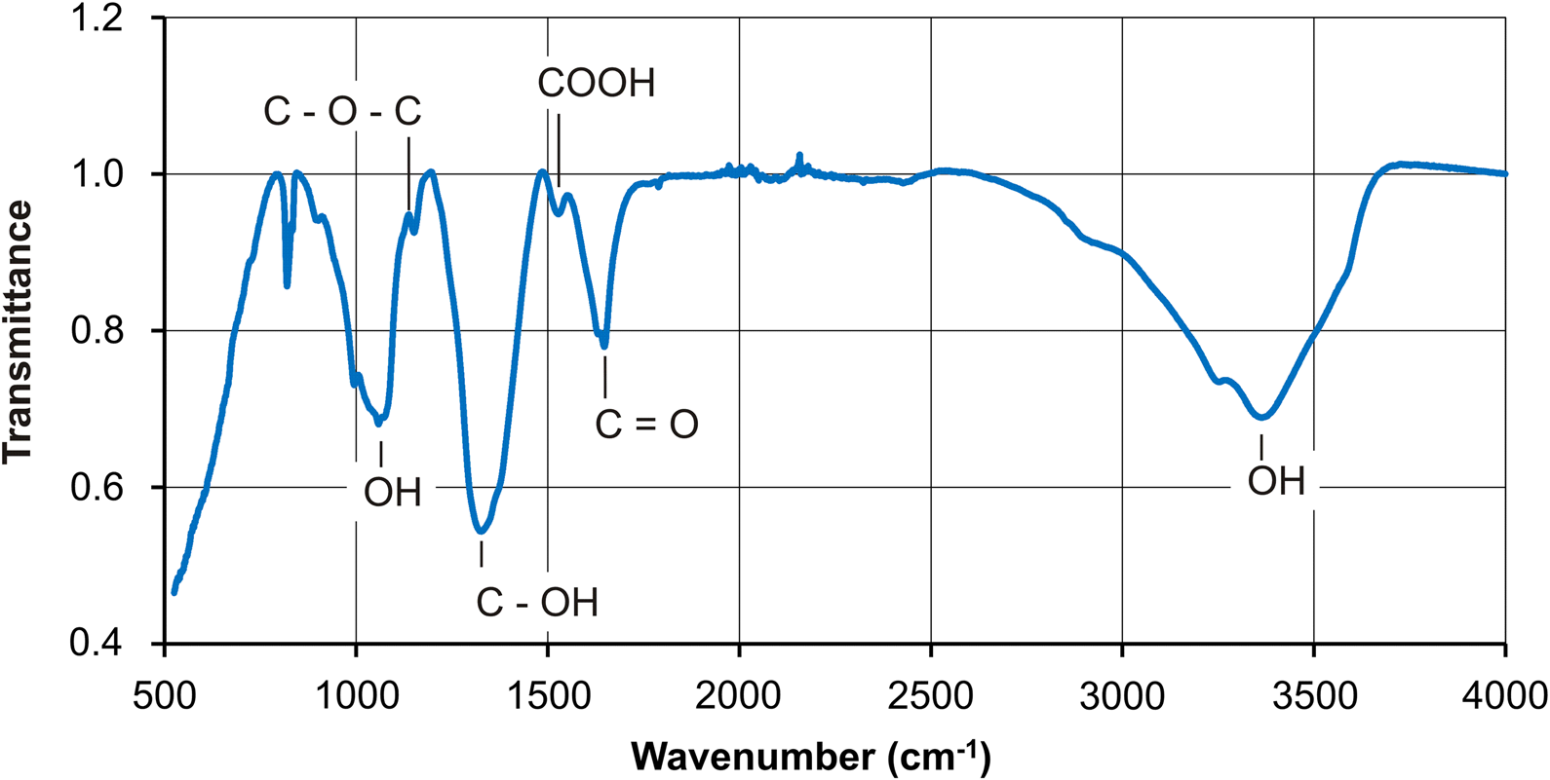
FT-IR spectrum of graphene oxide (GO)

**Fig. 8:**
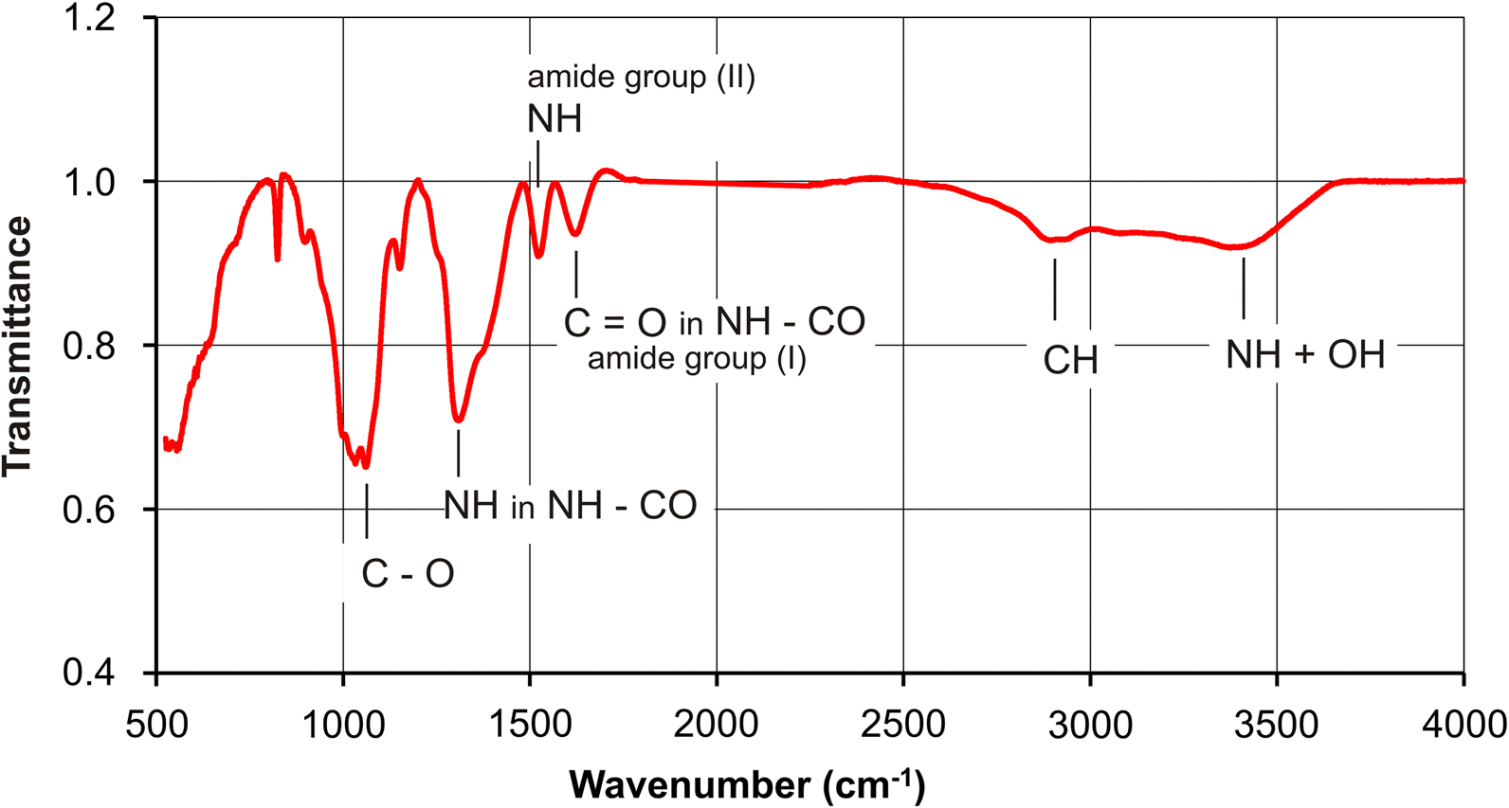
FT-IR spectrum for microcrystalline chitosan (MCCh) of deacetylation degree DD 97.7%

To confirm the potentiometric results in GO–Mg^2+^–MCCh, further FT-IR study was performed. The analysis examined the spectra obtained for GO, MCCh, GO–Mg^2+^–MCCh and MCCh–Mg at various pH values and degrees of MCCh deacetylation. Figures 7 and 8 present the FT-IR spectrum for the GO–MCCh complex at DD = 97.7%.

The FT-IR spectra (Fig. 7) for GO reveal the presence of a large number of functional groups such as OH, COOH, C=O, C–OH and C–O–C [17]. The FT-IR spectrum for MCCh shown in Fig. 8 reveals a wide band in the range 2500–3500 cm^−1^, overlapping the stretching vibrations of the OH and N–H groups. The C=O band in amide group I is present at 1624 cm^−1^, while the NH group of amide II can be observed at 1529 cm^−1^. A band corresponding to a CH group can be seen at 2935 cm^−1^. Another band at 1313 cm^−1^ corresponds to the NH group in NH-CO. The high intensity of the bands within the range 800–1200 cm^−1^ reveals the presence of a repeated pyranose ring structure present in the chitosan [3].

**Fig. 9:**
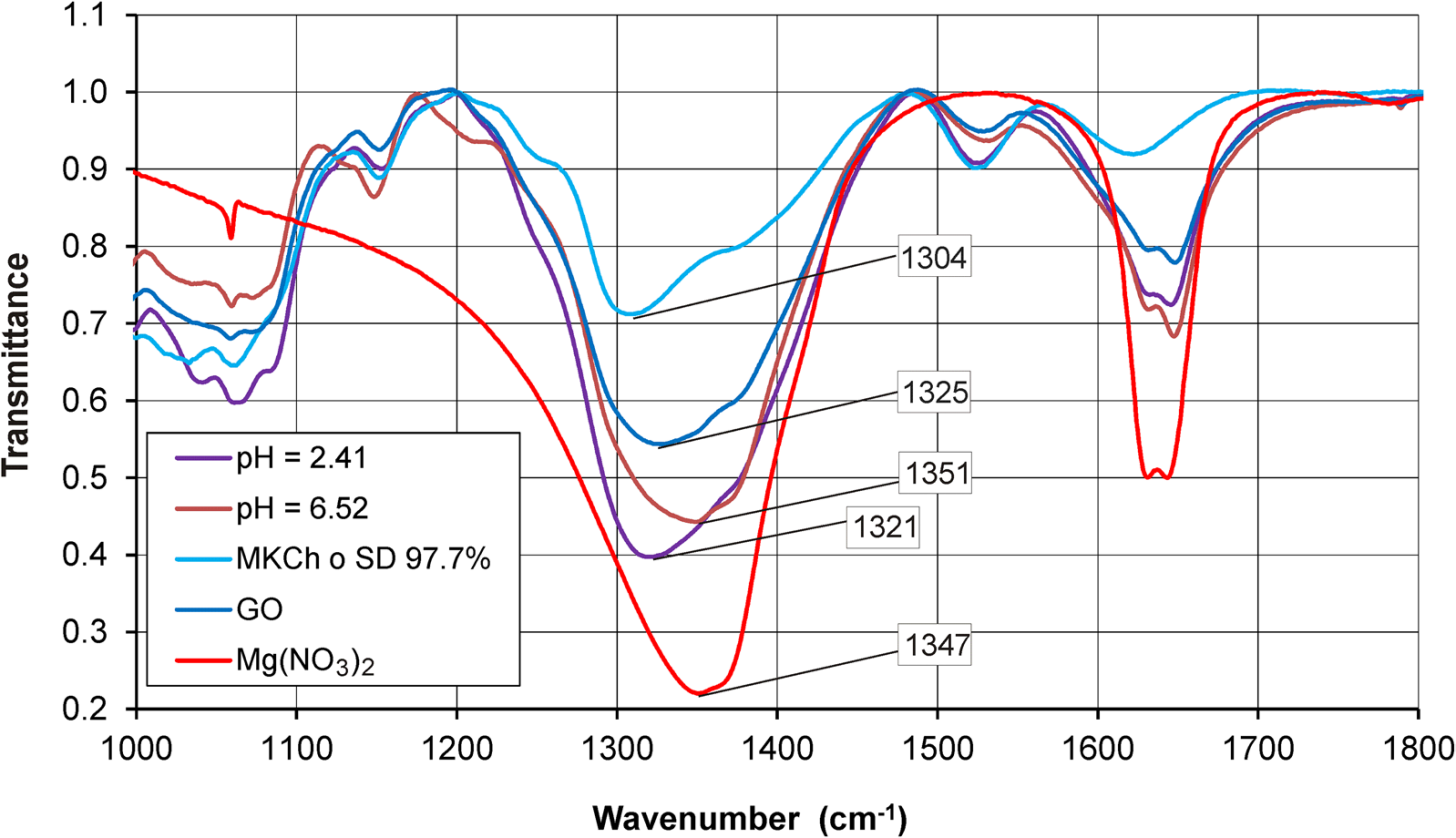
FT-IR spectrum for the GO–Mg–MCCh system of DD 97.7% at extreme pH values

**Fig. 10:**
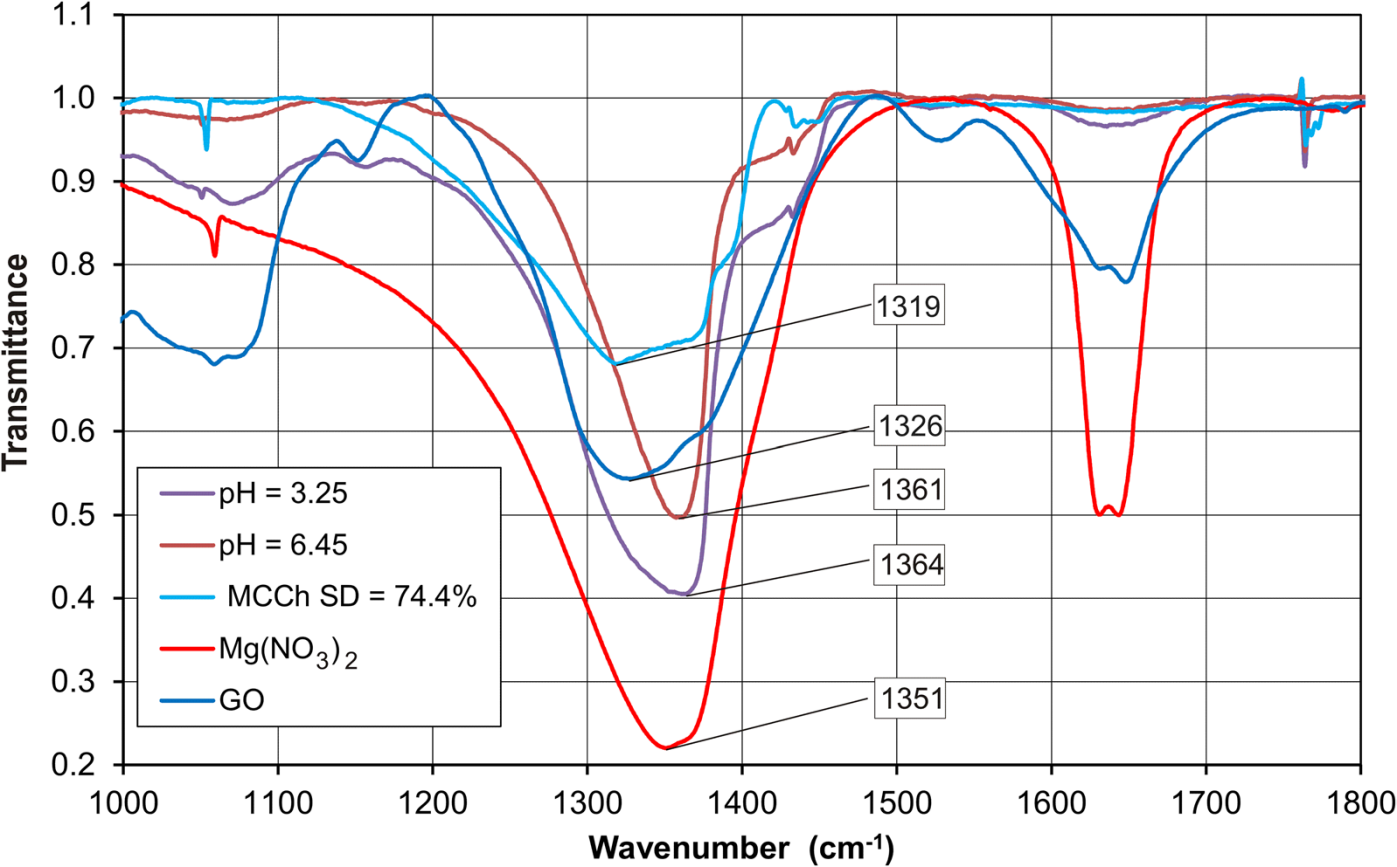
FT-IR spectrum for the GO–Mg–MCCh system of DD 74.4% at extreme pH values

The precise description of the individual spectra allowed for a better understanding of the interactions taking place in GO–Mg–MCCh [18]. As the presence of random pH values leads to poorly-differentiated spectra, a further analysis of the resulting curves were used for extreme pH values (Fig. 9). FT-IR analysis of GO–Mg–MCCh, at DD = 97.7% revealed displacement of the amide 1623 cm^−1^ II—NH band towards longer wave numbers, and its cleavage into two bands at 1633 cm^−1^ and 1648 cm^−1^, a change resulting from the interaction with the metal ion [19], and the band-shift of the chitosan acetyl NH group from 1304 cm^−1^ up to 1351 cm^−1^. When chitosan with a lower degree of deacetylation (DD = 74.4%) was applied, changes in the amide bands (amide I and II) were found to differ only in their intensity (Fig. 10). More pronounced changes were revealed for the absorption band of the NH of the acetyl group, of chitosan whose value moved from 1319 to 1361 cm^−1^. The above analysis indicates the existence of coordination interactions between GO, MCCh and the metal ion, in this case, the magnesium ion.

**Fig. 11:**
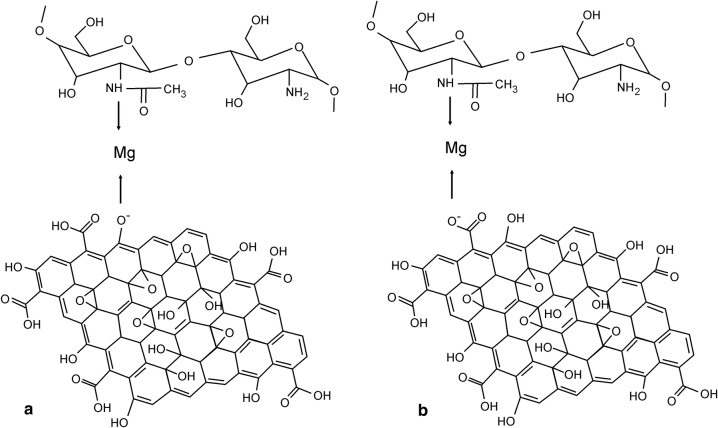
Proposed coordination modes in the GO–Mg–MCCh system of DD 74.4% and 97.7%, respectively. **a** Coordination via the OH group of graphene oxide, **b** via the COOH group of graphene oxide

Figure 11 proposes a model of coordination for GO–Mg–MCCh. The FT-IR analysis suggests that GO first binds to magnesium ions by the OH group, and then to the NH acetyl group of chitosan. It is also possible to combine GO and magnesium ions with COOH, and then with the NH of chitosan. It is known that in compounds containing a carboxyl group (COOH), comprising a carbonyl group C=O and the hydroxyl OH, the liquid particles are strongly associated due to the presence of intermolecular hydrogen bonds. Such bonds are very strong in both the liquid and solid states. In the formation of complex compounds, the carboxylate anion OCO-coordinates with the central ion in a different way. In the case of the analyzed system, it has a monodentate character [20].
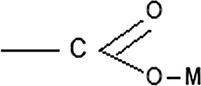

## Conclusions

Our findings confirm that donor–acceptor complexes can be formed for the systems GO–Mg^2+^ and GO–Mg^2+^–MCCh. Further analysis of our results indicates that type ML’ and ML’_2_ complexes are created in the GO–Mg ^2+^ system, and these are accompanied by MLL’ mixed complexes in the heteroligand system GO–Mg^2+^–MCCh. Our potentiometric pH studies confirm that the metal ion is coordinated by the oxygen of the carboxyl group and the oxygen of the hydroxyl group of GO; they also indicate that a mixed complex is formed by those groups, together with a nitrogen atom of chitosan, indicated by FT-IR spectroscopy to be an acetyl nitrogen. This greater understanding of the properties of GO–Mg^2+^–MCCh may improve knowledge of the application of a new generation of biomaterials, such as graphene oxide and microcrystalline chitosan, as carriers of active substances of biological importance.

## Methods

### Materials

**Fig. 12:**
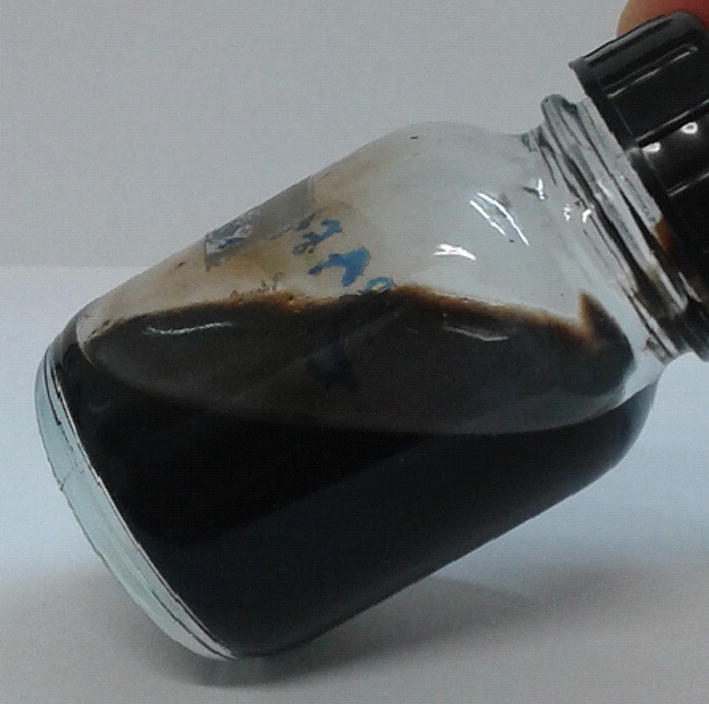
An aqueous suspension of graphene oxide GO

MCCh—microcrystalline chitosan (weight-average molecular weight = 326.0 and 380.0 kDa, Institute of Biopolymers and Chemical Fibers, Łódź, Poland) was used in the form of hydrogel of definite polymers contents (2.98 and 2.48 wt%) at two different degrees of deacetylation: 74.4, 97.7%. The DD degree, necessary to estimate the amount of –NH_2_ groups in the samples was determined by first derivative UV-spectrophotometry (1DUVS) according to Khor et al. [21]. Briefly, 0.01 mol L^−1^ Mg(II) stock solution was made up from Mg (NO_3_)_2_ × 6H_2_O, 0.2 mol L^−1^ KNO_3_ (POCh Gliwice), 0.1 mol L^−1^ from HNO_3_ 65% (Lach-Ner, Czech Republic). Carbonate-free 0.1000 ± 0.0003 M NaOH solution (Mallinckrodt Baker B.V.) was used as titrant. Two buffers: phthalate (0.05 mol L^−1^ potassium hydrophthalate + 0.05 mol L^−1^ KNO_3_, pH = 3.926 at 25 °C) and borax (0.01 mol L^−1^ Na_2_B_4_O_7_10H_2_O + 0.07 mol L^−1^ KNO_3_, pH = 9.10) were prepared at constant ionic strength 0.1 according to [22]. GO—graphene oxide, kindly supplied by Institute of Electronic Materials Technology, Warsaw, Poland, was used in the form of black aqueous suspensions (Fig. 12), molar mass of one mer M = 98.04 g/mol.

### Potentiometric titrations

A Molspin automatic titration kit (Newcastle upon Tyne, England) equipped with an OSH-10-10 combined electrode (METRON, Poland), and autoburette was used for EMF measurements. The total volume (alkali 0.1 mol L^−1^ NaOH carbonate-free, Malinckrodt Baker B. V.) in the Hamilton microsyringe of the automatic burette was 500 µL. The titration course was controlled by MOLSPIN software. For the potentiometric study of the GO–Mg^2+^ system, and of GO–Mg^2+^–MCCh, 4 mL of the sample was prepared, containing 0.031 mmol of GO, 0.030 mmol of 0.1 M nitric acid (V) and 0.0035 mmol of magnesium nitrate. The heteroligand system differed by the presence of only 0.028 mmol MCCh, so the molar ratio GO–MCCh was near 1:1. In turn, both the molar ratio of the ligand to the metal was about 8:1, which was intended to prevent early hydrolysis of the metal ion. All the experiments were carried out at 25.0 ± 0.1 °C and ionic strength 0.1 mol L^−1^ (KNO_3_). The cell was standardized with two buffers: pH = 3.926 and pH = 9.10 [23]. Before each titration, the electrode system was calibrated in the –log [H^+^] scale by strong acid–strong base titrations, 0.005 mol L^−1^ HNO_3_ was neutralized by 0.1 mol L^−1^ NaOH at temperature 25.0 ± 0.1 °C. The new parameters, differing from those obtained from the two-point cell standardization on pH, were then processed with Hyperquad2008 software to evaluate the overall, concentration formation constant: *β*_mll’h_ = [M*m*L*l*L’*l’*H*h*]/[M]^m^[L]^l^[L’]^l’^[H]^h^. Goodness-of-fit was tested by two parameters: *σ* (connected with the objective function) and χ^2^ statistics (test of randomness).

### FT-IR spectrophotometric measurements

Polymer films, with and without magnesium ions, were prepared for use in the FT-IR studies. The optimum L:M ratio was accepted as being the same as in the potentiometric measurements. Initially a portion of 0.030 mmol of nitric (V) acid was added to 0.028 mmol of MCCh to dissolve the ligand L. In other samples, after dissolving the ligand, 0.0035 mmol magnesium ions were also added. Further samples differed from the previous ones by addition of 0.031 mmol GO. By using 0.1 M NaOH, each sample was brought to a definite pH within the range 2.4–6.5. The aqueous slurry formed was put on a Teflon plate and left to dry at room temperature. The FT-IR spectra of the Mg^2+^ ion and GO were recorded using a Thermo Scientific Nicolet IS50 spectrophotometer, with all of the polymer film being used in the FT-IR measurements. A total of 64 scans were obtained. The spectral resolution was ± 2 cm^−1^.

## Abbreviations

DD: degree of deacetylation
FTIR: Fourier-transform infrared spectroscopy
L = MCCh: microcrystalline chitosan as ligand
GO: graphene oxide as ligand L’ magnesium ion as metal-M
L:M: the ligand-to-metal concentration ratio
*β*_*mll’h*_: [M_*m*_L_*l*_L_*l’*_H_*h*_]/[M]^*m*^[L]^*l*^[L’]^*l’*^[H]^*h*^ cumulative stability constant
*m*, *l*, *l*’, *h*: number of metals (central ions), ligands and protons, respectively; concentrations in square brackets are equilibrium concentrations
